# (Nitrato-κ*O*)bis­[5-(pyridin-2-yl)pyrazine-2-carbonitrile-κ^2^ *N* ^4^,*N* ^5^]silver(I)

**DOI:** 10.1107/S1600536811049221

**Published:** 2011-11-30

**Authors:** Fan Zhang, Yong-Li Yang

**Affiliations:** a Department of Chemistry, Capital Normal University, Beijing 100048, People’s Republic of China

## Abstract

In the mononuclear title complex, [Ag(NO_3_)(C_10_H_6_N_4_)_2_], two κ^2^ *N*:*N*′-chelating 5-(pyridin-2-yl)pyrazine-2-carbonitrile ligands surround the Ag^I^ atom, forming an N_4_O square-pyramidal coordination geometry with one nitrate anion bonding at the apical site. The two heterocyclic rings of the 5-(2-pyridin-2-­yl)pyrazine-2-carbonitrile ligand are almost coplanar [dihedral angle = 5.63 (8)°], and the two chelating ligands are in an *anti* relationship. The mononuclear units are inter­connected along [010] through C—H⋯O(nitrate) and C—H⋯N(cyano) inter­actions, forming an infinite chain. The mononuclear units are stacked along the *a* axis and inter­connected *via* inter­molecular π–π stacking inter­actions between adjacent pyridine and pyrazine rings [centroid–centroid distances = 3.984 (2) and 3.595 (3) Å], thus forming a three-dimensional supra­molecular structure.

## Related literature

For coordination complexes with pyridyl-based ligands, see: Dunne *et al.* (1997 ▶); Wang *et al.* (2009 ▶). For a related complex with 5-(2-pyridin-2-­yl)pyrazine-2-carbonitrile, see: Wang *et al.* (2010 ▶).
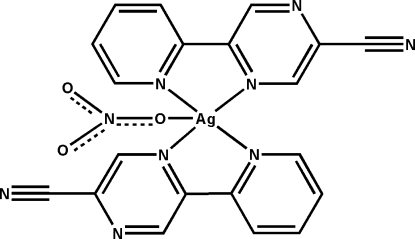


## Experimental

### 

#### Crystal data

[Ag(NO_3_)(C_10_H_6_N_4_)_2_]
*M* *_r_* = 534.26
Orthorhombic, 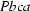

*a* = 14.000 (3) Å
*b* = 12.133 (2) Å
*c* = 23.832 (4) Å
*V* = 4048.4 (13) Å^3^
*Z* = 8
Mo *K*α radiation
μ = 1.04 mm^−1^
*T* = 293 K
0.44 × 0.35 × 0.25 mm

#### Data collection

Bruker APEXII CCD area-detector diffractometer
Absorption correction: multi-scan (*SADABS*; Bruker, 2007 ▶) *T*_min_ = 0.631, *T*_max_ = 1.000
27695 measured reflections
5029 independent reflections
3255 reflections with *I* > 2σ(*I*)
*R*_int_ = 0.037

#### Refinement

*R*[*F* ^2^ > 2σ(*F* ^2^)] = 0.034
*wR*(*F* ^2^) = 0.099
*S* = 1.03
5029 reflections
298 parameters
H-atom parameters constrained
Δρ_max_ = 0.46 e Å^−3^
Δρ_min_ = −0.28 e Å^−3^

### 

Data collection: *APEX2* (Bruker, 2007 ▶); cell refinement: *SAINT* (Bruker, 2007 ▶); data reduction: *SAINT*; program(s) used to solve structure: *SHELXS97* (Sheldrick, 2008 ▶); program(s) used to refine structure: *SHELXL97* (Sheldrick, 2008 ▶); molecular graphics: *SHELXTL* (Sheldrick, 2008 ▶); software used to prepare material for publication: *SHELXTL* and *PLATON* (Spek, 2009 ▶).

## Supplementary Material

Crystal structure: contains datablock(s) I, global. DOI: 10.1107/S1600536811049221/zq2135sup1.cif

Structure factors: contains datablock(s) I. DOI: 10.1107/S1600536811049221/zq2135Isup2.hkl

Additional supplementary materials:  crystallographic information; 3D view; checkCIF report

## Figures and Tables

**Table 1:** Hydrogen-bond geometry (Å, °)

*D*—H⋯*A*	*D*—H	H⋯*A*	*D*⋯*A*	*D*—H⋯*A*
C19—H19*A*⋯O1^i^	0.93	2.35	3.233 (3)	157
C11—H11*A*⋯O2^ii^	0.93	2.54	3.232 (5)	132
C13—H13*A*⋯N8^iii^	0.93	2.73	3.319 (3)	122

Symmetry codes: (i) 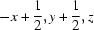; (ii) 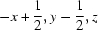; (iii) 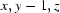.

**Fig. 1.**
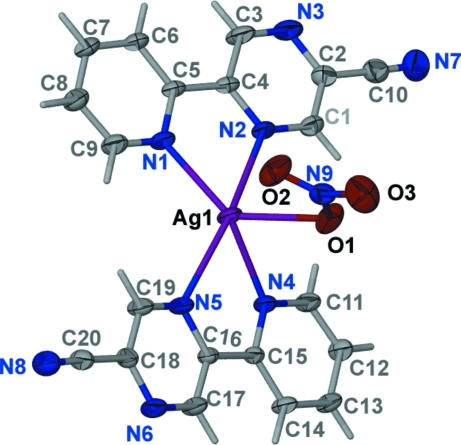
The atom-numbering scheme of the title compound. Displacement ellipsoids are drawn at the 30% probability level.

**Fig. 2.**
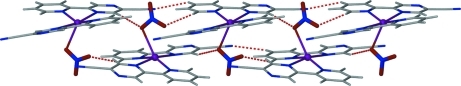
The infinite chain structure with C—H···O(nitrate) and C—H···N(cyano) interactions between the mononuclear units.The nitrate are shown as thick bonds for clarity.

**Fig. 3.**
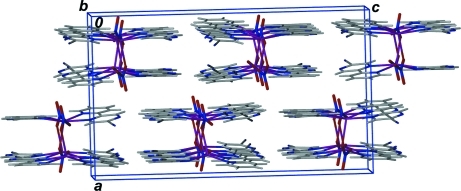
View down the b axis of the packing structure of the title complex. All non-covalent interactions are omitted for clarity.

## supplementary crystallographic information

### Comment

Pyridyl based ligands have attracted increasing attention because of their versatile linkage behavior and their artificial and controllable synthesis (Dunne *et al.*, 1997; Wang *et al.*, 2009). Recently, we reported a silver(I) complex derived from 5-(2-pyridyl)pyrazine-2-carbonitrile (Wang *et al.*, 2010). To make a further insight into the coordination chemistry of such a ligand featuring a 2-cyanopyrazinyl group at the 2-pyridyl carbon atom (Scheme 1), herein we present the structure of the new complex [Ag(C_10_H_6_N_4_)_2_(NO_3_)].

As shown in Fig. 1, in the mononuclear title complex two κ^2^*N:N* chelating 5-(2-pyridyl)pyrazine-2-carbonitrile ligands surround the Ag^I^ center to form a N4O-pyramidal coordination geometry with one nitrate bonding at the axial site. The Ag—N bond lengths lie within the range of 2.301 (2) - 2.579 (3) Å, with Ag1—N1 and Ag1—N4 slighty shorter than Ag1—N2 and Ag1—N5. These bond distances are comparable to those in [Ag(C_10_H_6_N_4_)_2_]BF_4_ (2.196 (2) - 2.685 (2) Å) reported by us recently (Wang *et al.* 2010). Furthermore, in the present case, the nitrate binds to the silver center with Ag1—O1 = 2.547 (3) Å. It is worth to note that a second O atom of the nitrate interacts with the silver center as shown by the Ag1—O2 distance of 2.800 (2) Å. Along the *b* axis, the mononuclear moieties are arranged with two adjacent ones around an inversion center. Indeed, C—H···O(nitrate) and C—H···N(cyano) interactions (Table 1) are found to link the mononuclear units together to form an infinite chain structure along the *b* axis (Fig. 2). Along the *a* direction, intermolecular π–π stacking interactions between adjacent pyridyl rings and pyrazinyl rings connect the mononuclear units together, forming a three-dimensional framework (Fig. 3). The distance between Cg1 (N4-N11-C12-C13-C14-C15) and Cg2^i ^(N5-N16-C17-N6-C18-C19) is 3.984 (2) Å, while that between Cg3 (N1-N5-C6-N7-C8-C9) and Cg4^ii ^(N2-C1-C2-N3-C3-C4) equals to 3.595 (3) Å (symmetry codes: i = –x, –y-1, –z+1; ii = –x+0.5, –y+1, z+1.5).

### Experimental

The 5-(2-pyridyl)-2-cyanopyrazine ligand was obtained commercially. The ligand (18.1 mg, 0.1 mmol) and AgNO_3_ (17 mg, 0.1mmol) were mixed and dissolved in 3 ml methanol, and then 1 ml acetonitrile was subsequently added to make the solution clear. After stirring at room temperature for 3 hours, the resulted solution was filtrated, and the clear solution was kept in air for about one week at room temperature to yield yellow block-like crystals (21.1.0 mg, 79% yield).

### Refinement

All H atoms were discernible in the difference electron density maps. Nevertheless, they were placed into idealized positions and allowed to ride on the carrier atoms, with C—H = 0.93 Å and *U*_iso_(H) = 1.2*U*_eq_(C).

### Crystal data

**Table:**

[Ag(NO_3_)(C_10_H_6_N_4_)_2_]	*Z* = 8
*M**_r_* = 534.26	*F*(000) = 2128
Orthorhombic, *P**b**c**a*	*D*_x_ = 1.753 Mg m^−^^3^
Hall symbol: -P 2ac 2ab	Mo *K*α radiation, λ = 0.71073 Å
*a* = 14.000 (3) Å	µ = 1.04 mm^−^^1^
*b* = 12.133 (2) Å	*T* = 293 K
*c* = 23.832 (4) Å	Block, yellow
*V* = 4048.4 (13) Å^3^	0.44 × 0.35 × 0.25 mm

### Data collection

**Table:**

Bruker APEXII CCD area-detector diffractometer	5029 independent reflections
Radiation source: fine-focus sealed tube	3255 reflections with *I* > 2σ(*I*)
graphite	*R*_int_ = 0.037
ω scans	θ_max_ = 28.3°, θ_min_ = 1.7°
Absorption correction: multi-scan (*SADABS*; Bruker, 2007)	*h* = −18→16
*T*_min_ = 0.631, *T*_max_ = 1.000	*k* = −12→16
27695 measured reflections	*l* = −31→31

### Refinement

**Table:**

Refinement on *F*^2^	Primary atom site location: structure-invariant direct methods
Least-squares matrix: full	Secondary atom site location: difference Fourier map
*R*[*F*^2^ > 2σ(*F*^2^)] = 0.034	Hydrogen site location: inferred from neighbouring sites
*wR*(*F*^2^) = 0.099	H-atom parameters constrained
*S* = 1.03	*w* = 1/[σ^2^(*F*_o_^2^) + (0.0413*P*)^2^ + 2.5136*P*] *P* = (*F*_o_^2^ + 2*F*_c_^2^)/3
5029 reflections	(Δ/σ)_max_ = 0.002
298 parameters	Δρ_max_ = 0.46 e Å^−^^3^
0 restraints	Δρ_min_ = −0.28 e Å^−^^3^

### Special details

**Table:**

Geometry. All esds (except the esd in the dihedral angle between two l.s. planes) are estimated using the full covariance matrix. The cell esds are taken into account individually in the estimation of esds in distances, angles and torsion angles; correlations between esds in cell parameters are only used when they are defined by crystal symmetry. An approximate (isotropic) treatment of cell esds is used for estimating esds involving l.s. planes.
Refinement. Refinement of F^2^ against ALL reflections. The weighted R-factor wR and goodness of fit S are based on F^2^, conventional R-factors R are based on F, with F set to zero for negative F^2^. The threshold expression of F^2^ > 2sigma(F^2^) is used only for calculating R-factors(gt) etc. and is not relevant to the choice of reflections for refinement. R-factors based on F^2^ are statistically about twice as large as those based on F, and R- factors based on ALL data will be even larger.

### Fractional atomic coordinates and isotropic or equivalent isotropic displacement parameters (Å^2^)

**Table:**

	*x*	*y*	*z*	*U*_iso_*/*U*_eq_	
Ag1	0.15007 (2)	0.51787 (2)	0.601301 (9)	0.06172 (11)	
N5	0.1257 (2)	0.6243 (2)	0.69391 (10)	0.0614 (7)	
N2	0.14122 (18)	0.4141 (2)	0.50864 (9)	0.0578 (6)	
C19	0.1326 (3)	0.7327 (3)	0.70324 (12)	0.0667 (9)	
H19A	0.1353	0.7807	0.6729	0.080*	
C16	0.1216 (2)	0.5589 (2)	0.73859 (11)	0.0483 (6)	
N6	0.1332 (2)	0.7114 (2)	0.80173 (10)	0.0705 (8)	
C15	0.1147 (2)	0.4379 (2)	0.72912 (11)	0.0462 (6)	
C20	0.1401 (3)	0.8922 (3)	0.76683 (13)	0.0628 (8)	
C18	0.1356 (2)	0.7745 (2)	0.75668 (12)	0.0539 (7)	
C1	0.1550 (2)	0.3072 (3)	0.49880 (13)	0.0640 (8)	
H1A	0.1696	0.2608	0.5286	0.077*	
N4	0.11379 (19)	0.4031 (2)	0.67615 (9)	0.0540 (6)	
N3	0.1261 (3)	0.3264 (3)	0.40160 (11)	0.0812 (9)	
C4	0.1200 (2)	0.4781 (3)	0.46515 (11)	0.0538 (7)	
C5	0.1057 (2)	0.5976 (3)	0.47527 (11)	0.0522 (7)	
N8	0.1425 (3)	0.9830 (3)	0.77670 (15)	0.0860 (11)	
C2	0.1481 (2)	0.2638 (3)	0.44573 (13)	0.0618 (8)	
C13	0.1112 (3)	0.2529 (3)	0.76347 (13)	0.0649 (9)	
H13A	0.1102	0.2027	0.7930	0.078*	
N1	0.1064 (2)	0.6321 (2)	0.52862 (9)	0.0609 (7)	
C17B	0.1266 (3)	0.6037 (3)	0.79202 (12)	0.0690 (10)	
H17A	0.1252	0.5561	0.8226	0.083*	
C11	0.1116 (3)	0.2943 (3)	0.66699 (12)	0.0643 (9)	
H11A	0.1100	0.2694	0.6301	0.077*	
C6	0.0916 (3)	0.6712 (3)	0.43157 (12)	0.0668 (9)	
H6A	0.0907	0.6461	0.3947	0.080*	
C14	0.1122 (3)	0.3652 (3)	0.77357 (12)	0.0606 (8)	
H14A	0.1112	0.3915	0.8102	0.073*	
C12	0.1117 (3)	0.2179 (3)	0.70918 (13)	0.0637 (8)	
H12A	0.1120	0.1430	0.7009	0.076*	
C10	0.1610 (3)	0.1471 (4)	0.43460 (15)	0.0709 (9)	
C9	0.0938 (3)	0.7393 (3)	0.53821 (13)	0.0724 (10)	
H9A	0.0941	0.7631	0.5753	0.087*	
C7	0.0789 (3)	0.7812 (3)	0.44284 (13)	0.0761 (11)	
H7A	0.0693	0.8311	0.4137	0.091*	
C8	0.0805 (3)	0.8168 (3)	0.49718 (14)	0.0752 (10)	
H8A	0.0728	0.8909	0.5060	0.090*	
N7	0.1715 (3)	0.0567 (3)	0.42452 (16)	0.0910 (10)	
C3	0.1130 (3)	0.4318 (3)	0.41204 (13)	0.0793 (11)	
H3A	0.0981	0.4780	0.3821	0.095*	
N9	0.3705 (3)	0.5055 (3)	0.60786 (12)	0.0663 (8)	
O1	0.3136 (2)	0.4333 (3)	0.61980 (14)	0.0984 (9)	
O2	0.3387 (3)	0.5884 (3)	0.58609 (14)	0.1118 (11)	
O3	0.4546 (3)	0.4916 (3)	0.61817 (18)	0.1278 (13)	

### Atomic displacement parameters (Å^2^)

**Table:**

	*U*^11^	*U*^22^	*U*^33^	*U*^12^	*U*^13^	*U*^23^
Ag1	0.0916 (2)	0.06265 (17)	0.03086 (12)	0.00339 (13)	−0.00402 (10)	0.01006 (10)
N5	0.105 (2)	0.0443 (14)	0.0350 (12)	−0.0033 (13)	0.0086 (12)	0.0042 (10)
N2	0.0754 (17)	0.0645 (17)	0.0334 (11)	0.0054 (13)	−0.0044 (11)	0.0051 (11)
C19	0.114 (3)	0.0477 (18)	0.0387 (14)	−0.0044 (17)	0.0097 (16)	0.0058 (13)
C16	0.0663 (18)	0.0449 (15)	0.0339 (13)	0.0023 (13)	0.0041 (11)	0.0039 (11)
N6	0.124 (3)	0.0491 (15)	0.0385 (12)	0.0014 (15)	0.0042 (14)	−0.0024 (12)
C15	0.0623 (17)	0.0428 (15)	0.0334 (12)	0.0014 (13)	0.0018 (11)	−0.0008 (11)
C20	0.090 (2)	0.050 (2)	0.0479 (16)	−0.0007 (17)	0.0101 (16)	−0.0005 (14)
C18	0.072 (2)	0.0433 (16)	0.0464 (15)	0.0000 (14)	0.0079 (13)	−0.0014 (13)
C1	0.080 (2)	0.069 (2)	0.0432 (16)	0.0067 (17)	−0.0080 (15)	0.0051 (15)
N4	0.0842 (17)	0.0446 (14)	0.0332 (11)	0.0018 (12)	0.0021 (11)	0.0007 (10)
N3	0.127 (3)	0.077 (2)	0.0402 (14)	−0.0038 (19)	−0.0035 (15)	−0.0020 (14)
C4	0.0628 (18)	0.066 (2)	0.0320 (13)	−0.0044 (15)	0.0005 (12)	0.0074 (13)
C5	0.0596 (17)	0.0646 (19)	0.0322 (12)	−0.0027 (15)	−0.0009 (12)	0.0089 (12)
N8	0.139 (3)	0.0503 (18)	0.069 (2)	−0.0001 (18)	0.0090 (19)	−0.0048 (15)
C2	0.068 (2)	0.068 (2)	0.0493 (16)	−0.0040 (17)	−0.0014 (14)	−0.0036 (15)
C13	0.104 (3)	0.0476 (18)	0.0434 (15)	−0.0059 (17)	−0.0042 (16)	0.0107 (13)
N1	0.0833 (18)	0.0636 (17)	0.0358 (12)	−0.0005 (14)	−0.0047 (12)	0.0078 (12)
C17B	0.128 (3)	0.0420 (17)	0.0372 (15)	0.0015 (18)	0.0034 (16)	0.0044 (13)
C11	0.108 (3)	0.0482 (18)	0.0373 (15)	−0.0021 (17)	−0.0001 (15)	−0.0038 (13)
C6	0.087 (2)	0.078 (2)	0.0353 (14)	0.0091 (19)	0.0002 (14)	0.0127 (15)
C14	0.097 (2)	0.0517 (18)	0.0330 (14)	−0.0058 (17)	−0.0010 (14)	0.0045 (13)
C12	0.097 (2)	0.0418 (17)	0.0527 (17)	−0.0037 (16)	−0.0004 (16)	−0.0017 (14)
C10	0.074 (2)	0.080 (3)	0.059 (2)	0.000 (2)	−0.0075 (16)	−0.0096 (19)
C9	0.106 (3)	0.068 (2)	0.0438 (16)	0.003 (2)	−0.0050 (17)	0.0031 (16)
C7	0.104 (3)	0.075 (3)	0.0491 (18)	0.014 (2)	0.0014 (18)	0.0234 (17)
C8	0.102 (3)	0.066 (2)	0.0574 (19)	0.0054 (19)	−0.0033 (18)	0.0120 (17)
N7	0.097 (3)	0.078 (2)	0.098 (3)	0.004 (2)	−0.022 (2)	−0.021 (2)
C3	0.134 (3)	0.069 (2)	0.0357 (15)	−0.003 (2)	−0.0082 (18)	0.0084 (16)
N9	0.083 (2)	0.065 (2)	0.0509 (16)	−0.0066 (15)	0.0031 (14)	−0.0043 (13)
O1	0.093 (2)	0.084 (2)	0.118 (2)	−0.0032 (17)	−0.0003 (17)	0.0475 (18)
O2	0.175 (3)	0.0582 (17)	0.102 (2)	−0.0110 (18)	−0.027 (2)	0.0188 (17)
O3	0.078 (2)	0.156 (3)	0.150 (3)	−0.005 (2)	−0.008 (2)	−0.026 (2)

### Geometric parameters (Å, °)

**Table:**

Ag1—N1	2.301 (2)	C4—C5	1.483 (4)
Ag1—N4	2.319 (2)	C5—N1	1.338 (4)
Ag1—N2	2.545 (3)	C5—C6	1.386 (4)
Ag1—O1	2.547 (3)	C2—C10	1.453 (6)
Ag1—N5	2.579 (2)	C13—C12	1.362 (4)
N5—C16	1.329 (3)	C13—C14	1.383 (4)
N5—C19	1.338 (4)	C13—H13A	0.9300
N2—C4	1.329 (4)	N1—C9	1.333 (4)
N2—C1	1.332 (4)	C17B—H17A	0.9300
C19—C18	1.371 (4)	C11—C12	1.368 (4)
C19—H19A	0.9300	C11—H11A	0.9300
C16—C17B	1.386 (4)	C6—C7	1.374 (5)
C16—C15	1.489 (4)	C6—H6A	0.9300
N6—C18	1.319 (4)	C14—H14A	0.9300
N6—C17B	1.331 (4)	C12—H12A	0.9300
C15—N4	1.331 (3)	C10—N7	1.132 (5)
C15—C14	1.379 (4)	C9—C8	1.369 (4)
C20—N8	1.128 (4)	C9—H9A	0.9300
C20—C18	1.450 (4)	C7—C8	1.365 (5)
C1—C2	1.373 (4)	C7—H7A	0.9300
C1—H1A	0.9300	C8—H8A	0.9300
N4—C11	1.338 (4)	C3—H3A	0.9300
N3—C3	1.317 (5)	N9—O3	1.215 (5)
N3—C2	1.333 (4)	N9—O2	1.216 (4)
C4—C3	1.388 (4)	N9—O1	1.218 (4)
			
N1—Ag1—N4	151.94 (10)	C6—C5—C4	121.8 (3)
N1—Ag1—N2	68.39 (9)	N3—C2—C1	121.7 (3)
N4—Ag1—N2	111.09 (9)	N3—C2—C10	116.1 (3)
N1—Ag1—O1	127.70 (10)	C1—C2—C10	122.2 (3)
N4—Ag1—O1	79.74 (9)	C12—C13—C14	118.2 (3)
N2—Ag1—O1	89.70 (10)	C12—C13—H13A	120.9
N1—Ag1—N5	107.92 (9)	C14—C13—H13A	120.9
N4—Ag1—N5	67.26 (8)	C9—N1—C5	117.8 (3)
N2—Ag1—N5	169.60 (9)	C9—N1—Ag1	119.6 (2)
O1—Ag1—N5	99.92 (10)	C5—N1—Ag1	121.9 (2)
C16—N5—C19	117.2 (3)	N6—C17B—C16	123.2 (3)
C16—N5—Ag1	113.13 (18)	N6—C17B—H17A	118.4
C19—N5—Ag1	128.68 (19)	C16—C17B—H17A	118.4
C4—N2—C1	117.6 (3)	N4—C11—C12	123.3 (3)
C4—N2—Ag1	113.5 (2)	N4—C11—H11A	118.4
C1—N2—Ag1	128.9 (2)	C12—C11—H11A	118.4
N5—C19—C18	121.4 (3)	C7—C6—C5	119.8 (3)
N5—C19—H19A	119.3	C7—C6—H6A	120.1
C18—C19—H19A	119.3	C5—C6—H6A	120.1
N5—C16—C17B	120.0 (3)	C15—C14—C13	119.8 (3)
N5—C16—C15	118.0 (2)	C15—C14—H14A	120.1
C17B—C16—C15	121.9 (2)	C13—C14—H14A	120.1
C18—N6—C17B	115.5 (3)	C13—C12—C11	119.1 (3)
N4—C15—C14	121.7 (3)	C13—C12—H12A	120.4
N4—C15—C16	117.2 (2)	C11—C12—H12A	120.4
C14—C15—C16	121.1 (2)	N7—C10—C2	178.2 (4)
N8—C20—C18	177.4 (4)	N1—C9—C8	124.5 (3)
N6—C18—C19	122.7 (3)	N1—C9—H9A	117.8
N6—C18—C20	115.9 (3)	C8—C9—H9A	117.8
C19—C18—C20	121.4 (3)	C8—C7—C6	119.4 (3)
N2—C1—C2	121.7 (3)	C8—C7—H7A	120.3
N2—C1—H1A	119.2	C6—C7—H7A	120.3
C2—C1—H1A	119.2	C7—C8—C9	117.5 (3)
C15—N4—C11	117.9 (2)	C7—C8—H8A	121.2
C15—N4—Ag1	122.46 (19)	C9—C8—H8A	121.2
C11—N4—Ag1	118.15 (19)	N3—C3—C4	123.7 (3)
C3—N3—C2	115.9 (3)	N3—C3—H3A	118.1
N2—C4—C3	119.4 (3)	C4—C3—H3A	118.1
N2—C4—C5	118.3 (2)	O3—N9—O2	123.7 (4)
C3—C4—C5	122.3 (3)	O3—N9—O1	119.2 (4)
N1—C5—C6	120.9 (3)	O2—N9—O1	117.0 (4)
N1—C5—C4	117.3 (2)	N9—O1—Ag1	105.0 (2)
			
N1—Ag1—N5—C16	161.6 (2)	Ag1—N2—C4—C5	0.6 (3)
N4—Ag1—N5—C16	11.1 (2)	N2—C4—C5—N1	−5.9 (4)
N2—Ag1—N5—C16	94.1 (5)	C3—C4—C5—N1	174.7 (3)
O1—Ag1—N5—C16	−63.2 (2)	N2—C4—C5—C6	174.4 (3)
N1—Ag1—N5—C19	−30.4 (3)	C3—C4—C5—C6	−5.0 (5)
N4—Ag1—N5—C19	179.1 (3)	C3—N3—C2—C1	0.9 (6)
N2—Ag1—N5—C19	−98.0 (5)	C3—N3—C2—C10	179.0 (4)
O1—Ag1—N5—C19	104.7 (3)	N2—C1—C2—N3	−0.8 (5)
N1—Ag1—N2—C4	2.6 (2)	N2—C1—C2—C10	−178.8 (3)
N4—Ag1—N2—C4	152.4 (2)	C6—C5—N1—C9	−0.5 (5)
O1—Ag1—N2—C4	−128.7 (2)	C4—C5—N1—C9	179.8 (3)
N5—Ag1—N2—C4	73.6 (5)	C6—C5—N1—Ag1	−171.4 (2)
N1—Ag1—N2—C1	−177.5 (3)	C4—C5—N1—Ag1	8.9 (4)
N4—Ag1—N2—C1	−27.7 (3)	N4—Ag1—N1—C9	88.5 (3)
O1—Ag1—N2—C1	51.2 (3)	N2—Ag1—N1—C9	−176.9 (3)
N5—Ag1—N2—C1	−106.5 (5)	O1—Ag1—N1—C9	−105.2 (3)
C16—N5—C19—C18	0.2 (5)	N5—Ag1—N1—C9	13.4 (3)
Ag1—N5—C19—C18	−167.3 (3)	N4—Ag1—N1—C5	−100.8 (3)
C19—N5—C16—C17B	−1.4 (5)	N2—Ag1—N1—C5	−6.2 (2)
Ag1—N5—C16—C17B	168.0 (3)	O1—Ag1—N1—C5	65.5 (3)
C19—N5—C16—C15	−179.5 (3)	N5—Ag1—N1—C5	−175.9 (2)
Ag1—N5—C16—C15	−10.1 (3)	C18—N6—C17B—C16	−0.6 (6)
N5—C16—C15—N4	0.4 (4)	N5—C16—C17B—N6	1.7 (6)
C17B—C16—C15—N4	−177.7 (3)	C15—C16—C17B—N6	179.7 (3)
N5—C16—C15—C14	178.7 (3)	C15—N4—C11—C12	−0.9 (5)
C17B—C16—C15—C14	0.6 (5)	Ag1—N4—C11—C12	165.4 (3)
C17B—N6—C18—C19	−0.7 (5)	N1—C5—C6—C7	0.5 (5)
C17B—N6—C18—C20	178.4 (3)	C4—C5—C6—C7	−179.8 (3)
N5—C19—C18—N6	0.9 (6)	N4—C15—C14—C13	1.8 (5)
N5—C19—C18—C20	−178.1 (3)	C16—C15—C14—C13	−176.4 (3)
C4—N2—C1—C2	0.3 (5)	C12—C13—C14—C15	−0.7 (6)
Ag1—N2—C1—C2	−179.6 (2)	C14—C13—C12—C11	−1.0 (6)
C14—C15—N4—C11	−0.9 (5)	N4—C11—C12—C13	1.9 (6)
C16—C15—N4—C11	177.3 (3)	C5—N1—C9—C8	−0.2 (6)
C14—C15—N4—Ag1	−166.7 (2)	Ag1—N1—C9—C8	170.9 (3)
C16—C15—N4—Ag1	11.5 (4)	C5—C6—C7—C8	0.1 (6)
N1—Ag1—N4—C15	−97.5 (3)	C6—C7—C8—C9	−0.7 (6)
N2—Ag1—N4—C15	179.1 (2)	N1—C9—C8—C7	0.8 (6)
O1—Ag1—N4—C15	93.5 (3)	C2—N3—C3—C4	−0.5 (6)
N5—Ag1—N4—C15	−11.9 (2)	N2—C4—C3—N3	0.0 (6)
N1—Ag1—N4—C11	96.8 (3)	C5—C4—C3—N3	179.4 (4)
N2—Ag1—N4—C11	13.4 (3)	O3—N9—O1—Ag1	172.5 (3)
O1—Ag1—N4—C11	−72.3 (3)	O2—N9—O1—Ag1	−7.3 (4)
N5—Ag1—N4—C11	−177.7 (3)	N1—Ag1—O1—N9	39.1 (3)
C1—N2—C4—C3	0.1 (5)	N4—Ag1—O1—N9	−147.4 (3)
Ag1—N2—C4—C3	180.0 (3)	N2—Ag1—O1—N9	101.1 (2)
C1—N2—C4—C5	−179.3 (3)	N5—Ag1—O1—N9	−82.9 (2)

### Hydrogen-bond geometry (Å, °)

**Table:**

*D*—H···*A*	*D*—H	H···*A*	*D*···*A*	*D*—H···*A*
C19—H19A···O1^i^	0.93	2.35	3.233 (3)	157
C11—H11A···O2^ii^	0.93	2.54	3.232 (5)	132
C13—H13A···N8^iii^	0.93	2.73	3.319 (3)	122

Symmetry codes: (i) −*x*+1/2, *y*+1/2, *z*; (ii) −*x*+1/2, *y*−1/2, *z*; (iii) *x*, *y*−1, *z*.
